# A novel transient structure with phylogenetic implications found in ratite spermatids

**DOI:** 10.1186/1471-2148-13-104

**Published:** 2013-05-26

**Authors:** Lizette du Plessis, John T Soley

**Affiliations:** 1 Electron Microscope Unit, Department of Anatomy and Physiology, Faculty of Veterinary Science, University of Pretoria, Onderstepoort 0110, South Africa; 2 Department of Anatomy and Physiology, Faculty of Veterinary Science, University of Pretoria, Onderstepoort 0110, South Africa

**Keywords:** Ratites, Spermiogenesis, Phylogenetics

## Abstract

**Background:**

A novel transient structure was observed in the spermatids of three ratite species using transmission electron microscopy.

**Results:**

The structure first appeared at the circular manchette stage of sperm development, was most prominent during the longitudinal manchette phase and disappeared abruptly prior to spermiation. It was composed of regularly-spaced finger-like projections which were closely associated with the outer nuclear membrane, giving the nucleus a cogwheel-like appearance. The projections were approximately 30 nm long and 14 nm wide. Although a similar structure has been described in certain lizard and crocodile species, this is the first report of a similar structure in the developing spermatids of birds.

**Conclusions:**

The potential value of non-traditional characters, such as spermiogenesis and sperm ultrastructure, as phylogenetic markers has recently been advocated. The morphologically unique structure found in ratite spermatids provides additional evidence of a possible phylogenetic link between the reptiles and birds. It also endorses the basal positioning of the ratites as a monophyletic group within the avian phylogenetic tree.

## Background

Phylogenetic relationships, both within the class Aves and also between birds and reptiles, remain controversial. Morphological and molecular studies support the concept that the avian phylogenetic tree consists of two major nodes, namely the Palaeognathae, which include the orders Struthioniformes (ostrich, rhea, emu, cassowary, kiwi) and Tinamiformes (tinamous), and the Neognathae to which all other bird species belong [1,2]. Traditionally, the ratites and the tinamous are placed at the base of the avian phylogenetic tree and as such are considered to be the most “primitive” living birds [3-5]. This view has been supported by morphological data as well as by nuclear and mitochondrial DNA sequencing [6-10]. However, other studies using similar techniques have yielded contrasting evidence which suggests that the Passeriformes may in fact be the oldest lineage of modern birds [11-13]. This contradictory situation is further complicated by the observation that the method of data analysis can influence the allocation of phylogenetic relationships [14,15].

Recently, the value of so-called non-traditional characters such as sperm ultrastructure and morphological aspects of spermiogenesis have been proposed as additional methods of indicating phylogenetic relationships [16-18]. Although spermiogenesis has been described in a variety of non-passerine bird species [19] including ratites [20-24], no morphological characters reflected during this process have, to date, been used to assist in assigning phylogenetic relationships.

This study describes the morphology of a unique, transient cytoplasmic structure that manifests during spermiogenesis in three ratite species, namely, the ostrich (*Struthio camelus*), rhea (*Rhea americans albisceu*s) and emu (*Dromaius novaehollandiae*). The possible function of the structure and its significance as a non-traditional phylogenetic character are discussed.

## Methods

The testes of 10 sexually mature and active emus (*Dromaius novaehollandiae*) and ostriches (*Struthio camelus*), respectively, were collected during the breeding season following slaughter at commercial abattoirs. Processed, resin-embedded testes samples of a rhea (*Rhea americans albisceu*s) were kindly supplied by Dr. David M. Phillips.

Small blocks of tissue were removed from the emu and ostrich testes and immediately fixed for 24 hours at 4°C in 4% glutaraldehyde buffered with 0.13 M Millonig’s phosphate buffer, pH 7.4. Samples were post-fixed for 1 hour in 1% similarly buffered osmium tetroxide and routinely prepared for transmission electron microscopy (TEM) [24,25]. Ultrathin sections were viewed in a Philips CM10 transmission electron microscope (Philips Electron Optical Division, Eindhoven, The Netherlands) operated at 80 kV. The resin samples of rhea testes had been fixed and processed as previously described [20] and were sectioned and stained as indicated above.

The linear dimensions of the individual projections forming the structure (length, width and distance between the projections) were determined using TEM images. A total of 45 projections was measured for each species. The measurements were processed using the Soft Imaging System iTEM software (Olympus, Műnster, Germany) and expressed as the average ± SD.

## Results and discussion

**Figure 1:**
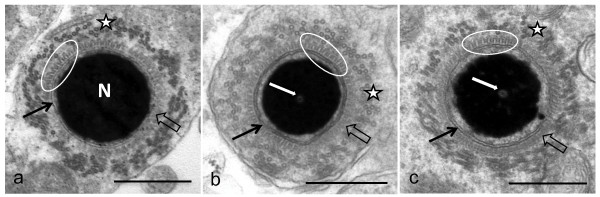
**Transverse sections of late stage spermatids in (a) the emu, (b) ostrich and (c) rhea.** Note the prominent longitudinal manchette microtubules (stars), the finger-like projections (encircled) closely associated with the outer nuclear membrane (black arrows) and the condensed chromatin of the nucleus (N). Individual manchette microtubules are closely aligned with, but not attached to, the finger-like projections. In some regions the projections appear as a fuzzy moderately electron-dense layer due to the plane of section (block arrows). The perforatorium typical of ostrich and rhea spermatids is visible within the nucleus (white arrows). Bar = 0.5 μm.

**Figure 2:**
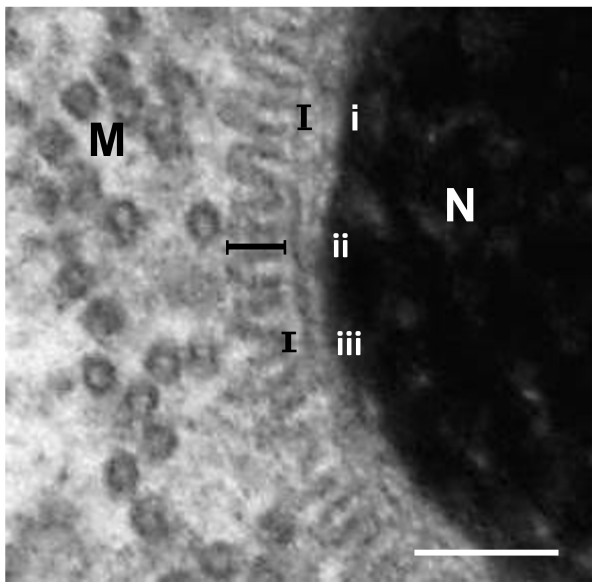
**High magnification of the finger-like projections observed in a transverse section of a late stage emu spermatid.** The average width, length and distance between projections are indicated by (i), (ii), and (iii). Nucleus (N); Manchette microtubules (M). Bar = 0.1 μm.

**Figure 3:**
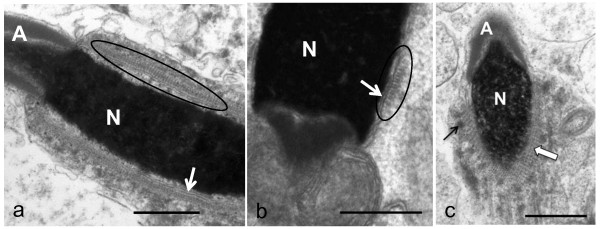
**Longitudinal sections through (a) emu and (b) rhea spermatids.** Note the prominence of the projections in the area immediately below the acrosome-nuclear shoulder in (**a**), but also at the base of the nucleus in (**b**) (encircled). (**c**) Oblique section of an emu spermatid demonstrating the typical lattice pattern (block arrow) formed by the projections. The nuclear chromatin (N) is in the form of coarse granules, indicating an earlier stage of spermatid development than that illustrated in (**a**) and (**b**). A series of cytoplasmic densities is apparent in the vicinity of the manchette microtubules (black arrow). Acrosome (A), nuclear membrane (white arrows). Bar = 0.5 μm.

Late-stage elongated spermatids in each of the three ratite species were characterised by the presence of a longitudinal collection of microtubules, the longitudinal manchette, surrounding the nucleus. The nucleus itself was composed of a dense mass of condensed chromatin closely enveloped by the nuclear membrane. In these spermatids, the zone of cytoplasm between the manchette microtubules and the nuclear membrane was occupied by a continuous array of small, regularly-positioned, finger-like projections which appeared to emanate from the cytoplasmic surface of the nuclear membrane. In transverse section the uniform and symmetrical arrangement of the projections and their close association with the nuclear membrane gave the nucleus a characteristic “cogwheel-like” appearance (Figures 1 and 2). While individual microtubules of the manchette were frequently linked by short, filamentous connections, there were no obvious connections between the microtubules of the longitudinal manchette and the finger-like projections. The observed morphology of the collective projections varied with the plane of section. In true transverse (Figure 1) or longitudinal sections of developing spermatids (Figure 3a,b) the projections manifested as typical finger-like structures. In oblique sections they adopted the form of a hazy zone of moderately electron-dense material (Figure 1) or as an array with a distinct lattice pattern (Figure 3c). The projections were identical in appearance when viewed in transverse or longitudinal sections of developing spermatids. Since the projections were visible in all profiles at this particular stage of spermatid development, it was concluded that they covered the entire surface of the nucleus. On average, the projections measured 29.75 ± 4.2 nm, 30.87 ± 4.09 nm and 31.7 ± 5.44 nm in length for the emu (n = 45), ostrich (n = 45) and rhea (n = 45), respectively. The width of the projections (measured at the base close to the nuclear membrane) was 13.36 ± 2.45 nm in the emu, 13.13 ± 1.68 nm in the ostrich, and 14.68 ± 2.61 nm in the rhea. The average distance between projections was 19.59 ± 4.4 nm in the emu, 19.49 ± 2.03 nm in the ostrich and 20.71 ± 4.39 nm in the rhea. In more advanced late stage elongating spermatids, the array of projections was observed to disappear leaving only a zone of flocculant material between the nuclear membrane and the manchette microtubules. This transition was abrupt.

The presence of transient nuclear membrane–associated projections during spermiogenesis in ratites has not been reported in any other avian species. The corresponding zone of cytoplasm of all other non-passerine species studied is occupied only by a layer of fine flocculant material [20,21,24,26-36]. The observed projections closely resemble structures previously reported in various lizard species [37-42] as well as in the Caiman (*Caiman crocodiles*) [43] and Nile crocodile (*Crocodylus niloticus*; personal observations). However, no morphological data other than the length of the “filaments” in the Caiman crocodile [43] have been presented. Based on TEM observations, the present study revealed that the projections were short, evenly spaced, peg- or finger-like extensions closely associated with the outer (cytoplasmic) face of the nuclear membrane. The projections were approximately 30 nm in length in the ratites compared to the 40 nm recorded in the Caiman crocodile [43]. Whether these observations point to inter-species variations, or simply reflect differences in technique and/or calibration of equipment, could not be determined. It is clear from this study that the nuclear membrane associated structure was not an artifact as it was present in all ratites examined (n = 21) despite differences in the preparation protocols [20,24,25]. Additionally, using the same technique, the structure was absent in closely related galliform and anseriform birds.

Various terms have been used to describe this transient structure. Examples include short filaments forming a “hairy headband” [43], radial projections or trabeculae associated with the nuclear membrane [37,40-42], bristles [44] and “saturations” originating from the nuclear envelope [38]. The use of these different and inconsistent terms accentuates the lack of detailed morphological information that is available. In lizards [37-42], and in both the Caiman [43] and Nile crocodiles, the projections appear to be restricted to the posterior aspect of the nucleus in the region just below the acrosome/nuclear shoulder, thus prompting the use of the term “hairy headband” [43]. In contrast, at the height of its development in the emu, ostrich and rhea, the projections were observed to cover the entire surface of the elongating spermatid nucleus, although they more obvious at the nuclear shoulder.

None of the earlier reports on reptiles provide evidence as to the possible nature of the “structure” other than to note the close connection of the projections with the cytoplasmic face of the nuclear membrane [37-39,41,42]. Similarly, its function remains enigmatic and its role during spermiogenesis is open to speculation. Ferreira and Dolder [42] were of the opinion that it connects the manchette microtubules to the nucleus, but failed to suggest a possible reason for such an association. Earlier reports noted a similar connection between the manchette microtubules and the “radial nuclear projections” [37,39], leading to the suggestion that they functioned together to anchor this region of the developing spermatid in order to facilitate tapering of the nucleus and its embedding within the surrounding Sertoli cell cytoplasm [39]. Al-Haji *et al*. [40] and Vieira *et al.*[41] also speculated that the projections anchor the manchette microtubules. Although the staggered arrangement of the longitudinal manchette placed a few individual microtubules close to the finger-like projections, no obvious morphological connection was observed between the two structures in ratites. No function has been ascribed to this “structure” in crocodiles [43].

Based on the parallel development of the “structure” and the manchette microtubules observed during spermiogenesis in the emu, ostrich and rhea, the proposed microtubule anchoring role of the projections is a plausible hypothesis. Classically, in mammals a sub-plasmalemmal density, the nuclear ring, forms posterior to the base of the acrosome from where the microtubules of the longitudinal manchette originate. The manchette has been widely implicated in mammals [45-50] and birds [24,26,27,29,32-34] in nuclear shaping/elongation and the caudal displacement of excess spermatid cytoplasm. Besides being anchored by the nuclear ring, a number of studies have identified linkers between individual microtubules of the manchette [24,49,51] which presumably gives extra stability to the microtubular array. Russell *et al*. [49] also reported on the existence of linkers between the manchette and the nucleus in rodents and speculated that they served to maintain a defined position of the manchette in relation to the nucleus. It is tempting to suggest that a similar situation occurs in ratites, particularly as a nuclear ring is not present, and that the projections serve as the link between the nucleus and the manchette. Although the projections appear to emanate from the outer face of the nuclear membrane in ratites, no obvious morphological connection between them and the manchette microtubules has been observed. This is clearly illustrated at the base of the nucleus where the projections point away from the manchette microtubules. In ratites the projections cover the entire surface of the nucleus whereas in lizards and crocodiles they are limited to the nuclear surface immediately beneath the acrosome. If, as suggested for reptiles [42], a link exists between the nucleus and manchette via the projections, it would appear as if the projections in these species fulfill a similar role to that of the nuclear ring in mammals.

The distribution of the projections throughout the spermatid nucleus in ratites and their lack of obvious contact with the manchette microtubules may indicate an alternative role, namely that of nuclear stabilization. Elongation of the nucleus in ratites is striking in comparison to the situation in mammals and in these avian species it may be necessary to support or stabilize the relatively long and thin developing nucleus. In this capacity the projections would act as a form of scaffolding, particularly during the period of translocation of spermatid cytoplasm (longitudinal manchette stage) when they are maximally developed. The sudden disappearance of the projections at the height of nuclear elongation (head shaping and chromatin condensation completed) would support this proposed function. The scattered distribution of the projections in the form of small pockets during earlier stages of nuclear transformation may be necessary to ensure a degree of stability without sacrificing the flexibility required for morphological transformation. However, it is not clear why ratite spermatids would require nuclear scaffolding when it is absent in other non-passerine birds which display equally long nuclei, for example the chicken [30; personal observations] and duck [52]; personal observations] and severely restricted in lizards [42] and crocodiles [43]. It could be argued that the nuclear membrane-associated projections seen in ratites and some reptilian species simply reflect a conserved character (symplesiomorphy) that is no longer of functional significance. An accurate determination of the composition of the projections would assist in answering this perplexing question.

Despite the lack of clarity on its function, the specific and organized nature of the transient “structure” revealed in the present study would appear to represent an important morphological character that can be applied for the resolution of phylogenetic questions as suggested by Gribbins [18]. Although not based on cladistic evidence, the observation that the only non-passerine birds with this unique structural adaptation studied to date appear to be the emu, ostrich and rhea, would support the traditional view that the extant ratites form a monophyletic group [2,8,13,53-56]. This view is strengthened by the fact that studies on galliform birds such as the chicken [26,28,30]; personal observations], turkey [34], quail [32] and members of the anseriforms [33,57]; personal observations], two families closely linked phylogenetically to the ratites, have failed to reveal an equivalent structure. It would be essential to determine whether the remaining members of the Ratidae (cassowaries and kiwis) also reflect the “structure” during spermiogenesis and indeed whether it is present in developing spermatids of the tinamous, as recent molecular studies have placed the tinamou together with the ratites as a polyphyletic group [2,15,58,59].

The exclusive nature of the “structure” would also suggest that the ratites occupy a basal position in the avian phylogenetic tree as has been previously proposed [3,7,10,13,60]. The only other vertebrates reported to display this “structure” are the crocodiles [43] and certain lizard species [37-42,44]. This may be viewed as additional evidence that reptiles and birds share a common ancestor [7,11,61-64]. The birds, crocodiles and turtles are estimated to have branched from the Archosaurs approximately 272 million years ago (MYA), whereas lizards, together with snakes and the tuatara, derived from the Squamates approximately 230 MYA. As both the Archosaurs and Squamates evolved from early reptiles approximately 285 MYA [63], and as modern-day relatives of both groups share the transient structure, it is plausible that it represents an ancient character trait which has been conserved in certain extant species. Future studies on spermiogenesis in a wider variety of birds and reptiles may provide clarity in this respect.

There is further compelling evidence to support the basal positioning of the ratites outlined above. Comparative sequence analysis of the α-crystallin A chain, a major constituent of the vertebrate eye lens, suggested that the ratites represent the first offshoot of the avian line with the crocodiles, represented by the alligator, as the sister group of the birds [60]. Similarly, mitochondrial protein sequencing [11], as well as amino acid sequencing and X-ray diffraction studies on the filament-matrix of β-keratins in emu feathers and scales of a lizard claw [64], supports the avian (ratite)/crocodile link. Various other anatomical/morphological studies have also reinforced the close relationship between reptiles and birds [10,61,62,65], including similarities in sperm structure [17]. Additionally, the morphological similarity between medullary bone of ratites and *Tyrannosaurus rex* points towards a close association between dinosaurs (an extinct member of the Archosaurs) and ratites [66].

## Conclusion

It is proposed that the nuclear membrane-associated projections seen during spermiogenesis in ratites and certain reptiles represent an early reptilian feature reflected in members of both the Archosaurs and Squamates. Despite contrary reports on avian phylogeny proposing various other birds, notably the passerines, to be the oldest offshoot of the avian stem [12,14,67-69], the existence of this unique structure in ratites strengthens the argument that these birds form a monophyletic group with a basal position within the avian phylogenetic tree. Morphological characters expressed during spermiogenesis therefore form an important complementary tool for the resolution of phylogenetic questions.

## Abbreviations

TEM: Transmission Electron Microscopy.

## Competing interests

The authors declare no competing interests.

## Authors’ contribution

LdP and JTS designed the study; LdP prepared the emu samples and JTS the ostrich samples; LdP collected the data; LdP and JTS analysed and interpreted the results as well as jointly wrote the paper. All authors read and approved the final manuscript.
